# Opioids: Modulators of angiogenesis in wound healing and cancer

**DOI:** 10.18632/oncotarget.15419

**Published:** 2017-02-16

**Authors:** Martina Ondrovics, Andrea Hoelbl-Kovacic, Daniela Alexandra Fux

**Affiliations:** ^1^ Department for Biomedical Sciences, Institute of Pharmacology and Toxicology, University of Veterinary Medicine Vienna, Veterinaerplatz, Vienna, Austria

**Keywords:** opioids, angiogenesis, signaling mechanisms, tumor vascularization, wound healing

## Abstract

Opioids are potent drugs that are widely used to control wound or cancer pain. Increasing evidence suggest that opioids mediate clinically relevant effects that go beyond their classical role as analgesics. Of note, opioids appear to modulate angiogenesis - a process that is critical in wound healing and cancer progression. In this review, we focus on pro- and anti-angiogenic facets of opioids that arise from the activation of individual opioid receptors and the usage of individual concentrations or application routes. We overview the still incompletely elucidated mechanisms of these angiogenic opioid actions. Moreover, we describe plausible opioids effects, which - although not primarily studied in the context of vessel formation - may be related to the opioid-driven processes of angiogenesis. Finally we discuss the use of opioids as an innovative therapeutic avenue for the treatment of chronic wounds and cancer.

## INTRODUCTION

Angiogenesis is the formation of new blood vessel out of pre-existing ones and includes endothelial cell activation, proliferation and chemotactic-driven migration. Angiogenesis has a pivotal role in embryonic development and growth [1], whereas desired and undesired effects are seen in adults. Beneficial effects of angiogenesis include efficient wound healing and the rescue of ischemic myocardium at early stages after myocardial infarction [2, 3]. Detrimental effects of angiogenesis appear in pathological processes such as macular degeneration, retinopathy, tumor growth, and metastasis [4–6].

Over recent years, the evidence has grown that opioids exceed their primarily known function as analgesic drugs and modulate wound healing and tumor progression [7, 8]. Opioids significantly influence wound closure or tumor growth by acting on endothelial cells and controlling angiogenesis. The review outlines the current knowledge of opioids’ action on angiogenic processes and discusses the potential exploitation of these effects for clinical use.

## THE OPIOID SYSTEM AND ITS ROLE IN ANGIOGENESIS

The endogenous opioid system represents a pivotal part of the innate central pain-relieving systems, which is operated by different opioid ligands and opioid receptors. Opioids are divided into “endogenous” and “exogenous” opiates, all having a potent analgesic effect. Endogenous opiates (Endorphins), such as beta-endorphin, Met-enkephalin, the endomorphins and the dynorphins are peptide hormones, which are generated in neurons of the nociceptive system and are released in pain and stress situations; exogenous opiates are non-peptidergic opioid receptor agonists. Prominent members of exogenous opiates are morphine, an alkaloid isolated from the poppy plant *Papaver somniferum*, and its semi-synthetic derivatives such as fentanyl, buprenorphine and oxycodone - classical analgesic drugs in clinical use [9, 10].

Opioid receptors belong to the family of G-protein coupled receptors (GPCRs) and are highly expressed in pain-modulating neurons of the central nervous system. Based on individual protein sequences and ligand selectivity, different opioid receptors can be distinguished. Besides the classical mu (μ-), delta (δ-) and kappa (κ-) opioid receptor types (further termed MOR, DOR and KOR), the opioid receptor family further includes the zeta (ζ-) receptor, also known as the Met-enkephalin receptor/opioid growth factor (OGF) receptor (further termed OGFR), and the nociception/orphanin stimulated FQ opioid-receptor like 1 (NOPr/ORL1) [11, 12]. Activation of opioid receptors initiates various intracellular signaling cascades which leads to the inhibition of adenylyl cyclase activity, modulation of ion conductance, transactivation of receptor tyrosine kinases, stimulation of phospholipase C, PI_3_K/AKT and the ras/raf/ERK1/2 signaling module [13–18]. Moreover, ligand binding also induces receptor phosphorylation, beta-arrestin recruitment and receptor internalization, which is believed to be a critical step in desensitization or termination of opioid receptor signaling [13]. The physiological function of neuronal opioid receptors became obvious by selective knockout of individual opioid receptor types in mice, which uncovered their special roles in nociception and mood disorders [19–22].

In recent years, opioid receptors - together with Met-enkephalin and dynorphin - were also found in endothelial cells during pre- and post-natal blood vessel development in mice and rats [23–25]. However, individual opioid receptors appear to elicit individual angiogenic effects as exemplified by the stimulation of OGFR, DOR and MOR in endothelial cells of the chorioallantoic membrane: whereas activation of OGFR by Met-enkephalin inhibits the formation of blood vessels by the opioid-exposed endothelial cells [26], stimulation of DOR (by Deltorphin I) or MOR (by Endomorphin-1 and -2) enhances blood vessel formation in the experimental setting [27]. In addition, inhibition of MOR by the antagonist naltrexone unexpectedly increased vessel formation by chorioallantoic membrane endothelial cells [26]. This finding suggests that MORs possess divergent roles in angiogenesis: whereas receptor stimulation by an opioid agonist leads to pro-angiogenic effect, the naltrexone-sensitive, constitutive (basal) receptor activity transmits anti-angiogenic effects [26, 28]. These differences may be explained by specific receptor conformations which are responsible for constitutive and agonist-induced activity of GPCRs [29, 30]. It has been shown that specific receptor conformations lead to the activation of individual G-proteins, which in turn are coupled to different intracellular signaling cascades [31]. It is therefore attractive to speculate that the constitutive active MOR conformation is coupled to inhibitory (anti-angiogenic), whereas agonist-occupied MORs are connected to stimulatory (pro-angiogenic) signaling pathways. Although further analysis of the divergent effects is missing, these findings yet indicate that opioid receptors may transmit both pro- and anti-angiogenic effects.

## OPIOID EFFECTS ON ISOLATED ENDOTHELIAL CELLS

Formation of new blood vessels during angiogenesis bases on endothelial cell proliferation, migration and tube formation. These processes are tightly coordinated by pro- and anti-angiogenic factors *via* stimulating respective cell surface receptors and signaling pathways in endothelial cells. A variety of pro-angiogenic factors have been identified during the last decades - the Vascular Endothelial Growth Factor (VEGF-A) and its splice variants VEGF_121_, VEGF_165_, VEGF_189_ being most potent [32, 33]. VEGF binds to and activates the receptor tyrosine kinases VEGF receptor-1 (VEGFR-1/Flt1) and VEGFR-2 (KDR/Flk-1) [34], which leads to the stimulation of JNK, ERK1/2, PI_3_K/AKT, and focal adhesion kinases. All of these VEGF/VEGFR-driven effector molecules are well-described regulators of endothelial cell survival, proliferation, cell migration, and thus vessel neo-formation [35].

### Stimulatory opioid effects on endothelial cells

A close interaction of the opioid system and VEGF-mediated angiogenesis is suggested by two major observations. (i) VEGF enhances the expression of MOR in endothelial cells. (ii) Morphine activates endothelial VEGF receptors including their associated signaling molecules AKT and ERK1/2 [36]. Gupta et al. [37] observed that treatment of endothelial cells (HDMEC; isolated from neonatal human foreskin) with VEGF_165_ as well as clinically relevant concentration of morphine cause cell proliferation and tube formation. Studies on endothelial cells derived from different tissues further confirmed a causal relationship of opioids and angiogenesis: proliferation and migration of dermal microvascular and retinal endothelial cells were enhanced upon morphine treatment [36, 38]. The mode by which morphine triggers these endothelial cell reactions is currently under debate. The classical signaling *via* binding to opioid receptors is rather unlikely as morphine-induced proliferation and tube formation cannot be blocked by the opioid-receptor antagonist naloxone [37]. In contrast, the endothelial morphine effects could be blocked by a VEGF receptor inhibitor [38]. This finding leads to the suggestion that morphine activates VEGF receptors and exploits their angiogenic signaling on endothelial cells. Detailed understanding of *how* morphine masters VEGF receptor activation is currently lacking. Activation of VEGF receptors may arise from two different mechanisms: one is VEGF-dependent, the other one is VEGF-independent. The growth factor-dependent mechanism bases on an activation of matrix metalloproteinases, which initiate VEGF receptor transactivation by the release of extracellular matrix-bound VEGF (“outside-in mechanism”). In contrast, the VEGF-independent mechanism triggers VEGF receptor activation through phosphorylation of the receptor protein by the intracellularly localized non-receptor tyrosine kinase c-Src [39]. The “outside-in mechanism” seems to play a minor role in morphine-induced VEGF receptor activation, as morphine was shown to prevent VEGF_121_ and VEGF_165_ release from stimulated endothelial cells [40]. Increasing evidence in turn supports the ligand-independent activation as c-Src was shown to be activated in endothelial cells upon morphine treatment [38]. Moreover, c-Src knock-down by siRNA prevented morphine-induced VEGF receptor activation, endothelial cell proliferation and tube formation. These findings indicate that the pro-angiogenic morphine effects result from c-Src-dependent VEGF receptor transactivation [38].

**Figure 1:**
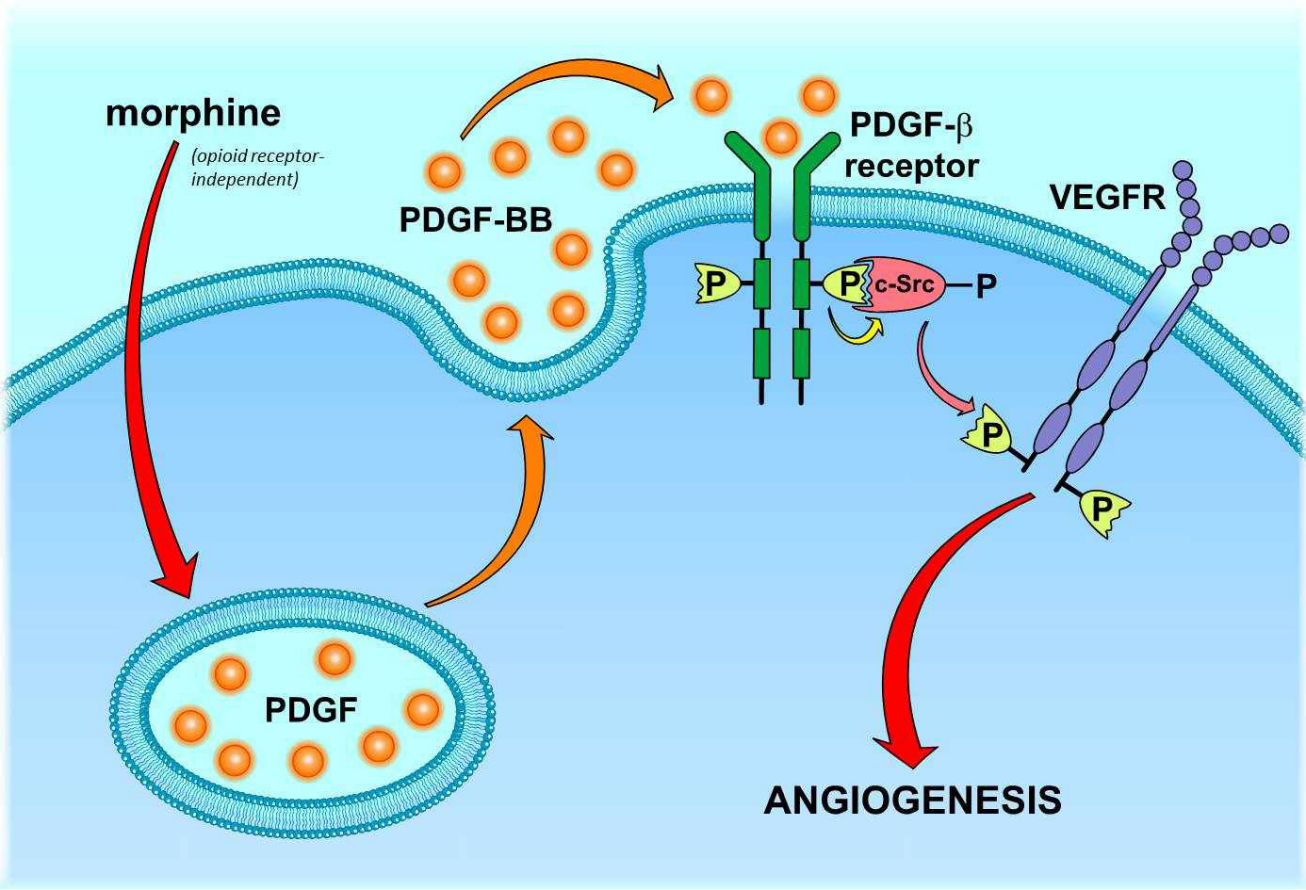
Proposed mechanism of morphine-mediated PDGFR/VEGFR co-activation in endothelial cells Morphine stimulates the release of PDGF-BB by an opioid-receptor-independent, yet unknown mechanism. Released PDGF-BB leads to an autocrine activation of endothelial PDGF-β receptors, which subsequently recruit and activate c-Src kinase. Stimulated c-Src kinase triggers phosphorylation and thus VEGF-independent activation of VEGF receptors (VEGFR).

This finding, however, raises the next question: *how* does morphine stimulate c-Src? A potential candidate stimulus is the platelet-derived growth factor (PDGF-BB). PDGF-BB was found to be released from morphine-stimulated endothelial cells [41]. Moreover, PDGF-BB is known to induce c-Src activation upon binding to PDGF receptors (PDGFR; [42, 43]. As PDGFRs were additionally found to be co-activated with VEGF receptors in morphine-treated endothelial cells [36], these facts suggest that morphine may induce VEGF receptor activation by stimulating the PDGF/PDGFR/c-Src signaling cascade (Figure 1). Considering the release of PDGF as the critical starting point of this scenario, it is still unclear how morphine induces this process in an opioid-receptor independent manner. It might be speculated that morphine triggers PDGF secretion by direct activation of G-proteins, which was identified to account for histamine release from morphine-stimulated mast cells [44]. However, the regulating processes of PDGF secretion as well as the identity of 454 non-redundant putative morphine off-targets are still obscure [45, 46]. Thus, the opioid-receptor independent signaling machinery behind morphine-induced PDGF release remains an unsolved question.

### Inhibitory opioid effects on endothelial cells

Opioids were also shown to inhibit the angiogenic activity of endothelial cells [47–49]. In contrast to the aforementioned opioid receptor-independent morphine effects, stimulation of two specific opioid receptors types were identified to transmit anti-angiogenic effects on isolated endothelial cells. One is the vascular-selective μ3-opioid receptor, which was found to elicit the production of lethal amounts of nitrogen oxide (NO) after stimulation by a high dose of morphine [48, 50]. NO was further proven to have a causal role in morphine-induced death of endothelial cells, as inhibition of NO synthase by L-NAME rescued cells from apoptosis. Also, naloxone prevented cells from morphine-induced apoptosis [48] which underlines opioid receptor-dependent stimulation of NO synthase activity may counteract the angiogenic activity of endothelial cells.

The second opioid receptor type with anti-angiogenic properties is the KOR, which was shown to impair VEGF-induced endothelial cell responses. Stimulation of KORs by U50,488H or Nalfurafine reduced VEGR expression levels in endothelial cells and significantly hampered VEGF-induced migration and tube formation [51]. These effects were reversed by the KOR antagonist nor-BNI or siRNA-mediated KOR knock-down, indicating that the regulation of VEGF receptor expression is indeed KOR-dependent. In line, *Kor* knockout mice show enforced expression of VEGF receptors in endothelial cells [51]. The causative mechanisms of these findings have not been investigated so far, but it might be envisioned that KORs affect transcription factors, promoter activity or mRNA stability controlling VEGF receptor protein expression.

## OPIOID EFFECTS ON PLUG VASCULARIZATION

In analogy to the conflicting results obtained from isolated endothelial cells, morphine also exerts different effects on *in-vivo* vessel formation, which was revealed by Matrigel plug assays. In this approach, Matrigel plugs are implanted subcutaneously into mice and monitored for vascularization that is indicative for chemotactic endothelial cell migration, proliferation and vessel formation. When morphine was incorporated into the plug matrix, plugs were significantly stronger vascularized than morphine-free controls [37] - suggesting morphine a chemoattractant for invading endothelial cells as it has been reported for monocytes and neutrophils [52]. Vascularization of morphine plugs was not affected by naloxone, which suggests morphine-triggered chemotaxis does not depend on opioid receptor stimulation [53]. The processes of chemotaxis are highly complex and involve various signaling mechanisms and pathways, which might represent potential morphine off-target. In monocytes, for instance, chemotaxis is facilitated by activated potassium channels [54]. Interestingly, morphine was found to stimulate ATP-dependent potassium currents by an opioid-receptor independent mechanism in hepatocytes [55] As ATP-dependent potassium channels are also expressed in endothelial cells [56], morphine might be assumed to stimulate chemotaxis *via* direct activation of potassium currents. Nevertheless, the precise factors accounting for morphine-stimulated chemotaxis are still elusive.

In a setting where morphine was applied systemically *via* intraperitoneal or subcutaneous injections for a long time (chronic application), plug vascularization was impaired. In these studies, implanted plugs contained lipopolysaccharide (LPS) or the angiogenic factors VEGF and FGF. Although all of these “plug ingredients” are potent inducers of angiogenesis, plug vascularization was significantly reduced upon long-term, systemic morphine application [47, 57, 58]. Thus, systemically applied morphine seems to prevent LPS- and VEGF/FGF-mediated endothelial cell invasion and subsequent vessel formation. A first mechanistic insight was given by the finding that vascularization of VEGF plugs was restored in MOR deficient mice, which revealed the inhibitory morphine effect depends on opioid receptor stimulation [53]. Although further insights are lacking, alternative morphine effects may be considered to enlighten the processes of impaired plug vascularization. Vascularization of Matrigel implants is facilitated by endothelial cells which are attracted and finally stimulated by a gradient of angiogenic factors. In the case of VEGF plugs, endothelial cells are directly triggered by plug-released VEGF. The activity of endothelial cells to detect and follow the VEGF gradient is enhanced by the Endothelial Cell-Specific Chemoattractant Receptor (ECSCR), also known as the Endothelial Cell-Specific Molecule 2 (ECSM2), which improves the sensitivity and responsiveness of VEGF receptors towards VEGF stimulation [59, 60]. Expression of ECSCR is increased by inflammatory processes [61], so that local inflammation, which arises from plug injection - as indicated by the enrichment of inflammatory immune cells in the plug transplants [62] - strongly supports VEGF-mediated chemotactic activity of endothelial cells. Long-term, systemically applied morphine, however, has immune suppressive and anti-inflammatory effects [63]. It could be said that morphine affects endothelial cell chemotaxis and therefore VEGF plug vascularization through its anti-inflammatory effect, which probably hinders ECSCR expression and angiogenic signaling in endothelial cells.

Plug-released LPS triggers vessel formation by direct stimulation of the angiogenic Toll-like receptor/TRAF6 signaling pathway in endothelial cells [64] and by attracting and stimulating VEGF-producing macrophages [65]. Both processes may represent hypothetical morphine targets. A recent study reported that morphine-stimulated MORs on macrophages counteract LPS-induced miR-146a expression [66]. As miR-146a triggers VEGF synthesis [67], morphine-exposed macrophages may fail to provide a sufficient VEGF gradient which is required for endothelial cell chemotaxis and activation. Moreover, chronic morphine was revealed to inhibit LPS-induced activation of TRAF6 signaling in macrophages by inducing miR-124 expression [68]. If this effect would also occur in endothelial cells, morphine could hamper LPS-stimulated vessel formation by blocking the angiogenic TRAF6 signaling in endothelial cells.

The lack of FGF-plug vascularization may be also explained by a morphine effect on an angiogenic FGF signaling partner. FGF-induced vessel formation requires the presence of integrins, which serve as direct FGF-binding receptors as well as signaling partner and enhancer of the FGF/FGF receptor signal complex [69]. A recent study has shown that long-term treatment of mice with morphine induces a reduction of integrin expression in neurons of the spinal cord [70]. If this morphine effect also occurs outside the central nervous system, it may be possible that impaired vascularization of FGF-plugs arises from a morphine-induced loss of integrins in angiogenic active endothelial cells.

## OPIOID EFFECTS ON WOUND VASCULARIZATION

Wound healing is a highly dynamic process, which requires vasodilatation, fibrin clot formation, infiltration of neutrophils, macrophages and lymphocytes, proliferation of fibroblasts, re-epithelization by stimulated keratinocytes, and tissue maturation. Each of these healing phases is supported by wound vascularization, which guarantees restoration of tissue oxygenation, nutrition supply and stimulation of wound-associated cells to fill-up and re-organize the wound space [71]. As opioid receptors have been discovered in wound-associated endothelial cells as well as in VEGF-expressing fibroblasts and keratinocytes, a prominent role of the opioid system in wound vascularization is suggested [72, 73].

### Positive effects on wound angiogenesis

**Figure 2:**
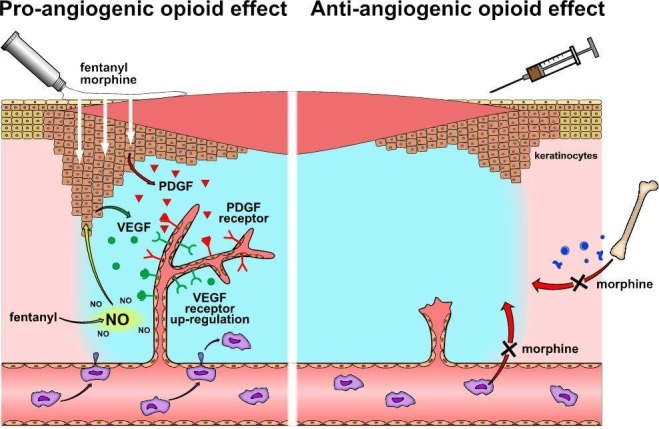
Wound angiogenesis under the influence of opioids Topically applied opioids (*left panel*) increase VEGF receptor expression and stimulate PDGF receptors in wound-associated endothelial cells. Activation of PDGF receptors might result from PDGF, which is released from opioid-stimulated keratinocytes. Fentanyl augments NO concentration in wounds, which triggers VEGF synthesis in keratinocytes. Released VEGF may activate angiogenic signaling of up-regulated VEGF receptors in wound-associated endothelial cells. In contrast, systemic morphine prevents wound vascularization by hindering macrophages from wound infiltration and recruitment of endothelial progenitor cells (*right panel*).

In an ischemic wound model, topically applied morphine, hydromorphone and fentanyl enhanced wound vascularization by inducing endothelial cell proliferation [74, 75]. As endothelial cells in opioid-exposed wounds showed an increased activation of PDGFR-β [74] and higher abundance of VEGFR-1 [75], the *in-vitro* observed endothelial PDGFR/VEGFR co-signaling [36] may also account for opioid-stimulated proliferation of endothelial cells *in-vivo*. As an additional, supportive effect, opioids could trigger endothelial cell proliferation *via* paracrine activation of PDGFR signaling that is facilitated by keratinocytes. Keratinocytes were identified to possess responsive opioid receptors [73, 76]. Moreover, keratinocytes represent the major source of cutaneous PDGF [77]. It may be speculated that in analogy to C6 neuronal cells [78], keratinocyte stimulation by opioids results in paracrine release of PDGF, which in turn acts on endothelial PDGFRs and promotes angiogenesis (Figure 2).

The contribution of opioid-induced NO synthesis in wound angiogenesis is currently under debate. As described above, increased NO concentrations *in-vitro* leads to endothelial cell death. In contrast, increase of NO levels *in-vivo* - as observed in fentanyl-exposed skin defects - is associated with an enhanced endothelial cell proliferation and accelerated wound healing [74]. The differences observed in *in-vitro* and *in-vivo* studies on NO effects may be explained by wound-specific mechanisms that counteract NO and lethal reactive nitrogen species (RNS). These mechanisms may include anti-oxidative tissue enzymes such as heme oxygenase, superoxide dismutase or glutathione peroxidase, which act as key players in wound healing and bring balance to NO-generated RNS [79]. In addition, NO prompts other, non-endothelial wound infiltrating cells, especially keratinocytes and fibroblast for VEGF synthesis - thereby protecting endothelial cells from apoptosis and increasing their proliferation [80, 81]. Considering this multifactorial *in-vivo* scenario, opioid-induced NO synthesis in wounds is conceivable to support vascularization and healing process after topical opioid application.

Rather unexpected, was the observation that an opioid receptor antagonist may have beneficial effects on wound vascularization. Topically applied naltrexone was found to enhance VEGF-expressing endothelial cells in wounds [82]. This observation raises the question by which mechanisms an opioid receptor antagonist may provide stimulatory effects. A possible route could be *via* the endothelial OGFR. Naltrexone is known to bind to OGFR and prevents its activation by endothelial Met-enkephalin. As OGFR signaling is reported to suppress proliferation of endothelial cells [24], binding of naltrexone to the OGFR is likely to promote vessel formation by counteracting the anti-angiogenic Met-enkephalin/OGFR signaling axis [24, 26]. As a concomitant effect, unbound Met-enkephalin - no longer able to bind OGFR - may stimulate a pro-angiogenic, naltrexone-insensitive opioid receptor. Such a scenario may be performed by DORs as the opioid receptor type is activated by Met-enkephalin and binds naltrexone only with low affinity [28, 83]. A pro-angiogenic signaling of stimulated DORs was already demonstrated for chorioallantoic vessels (see above) [27]. Thus, a Met-enkephalin stimulated DOR signaling can be envisioned for wound-associated endothelial cells and may explain naltrexone-enhanced angiogenesis.

### Negative effects on wound angiogenesis

In contrast to topical application, long-term application of morphine or morphine-sulfate *via* subcutaneous and intraperitoneal injections delayed vascularization and closure of an excisional skin injury in mice [47, 84]. Thus, a systemic morphine effect prevents the angiogenic activity of wound-associated endothelial cells. Wound vascularization is organized by vasculogenic cytokines and growth factors that stimulate adjacent endothelial cells (angiogenesis) and attract circulating endothelial progenitor cells for *de-novo* vessel formation (vasculogenesis) [85]. The lack of wound angiogenesis is in line with previous observations, in which systemic, long-term application of morphine hampered endothelial cell chemotaxis towards growth-factor containing Matrigel plugs (see above). Moreover, long-term systemic application of morphine led to a significant reduction of circulating endothelial progenitor cells [47], which also points out a failure in vasculogenesis. Martin et al. [58] showed in an independent study that systemically applied morphine reduces the migration activity of macrophages and wound infiltration (Figure 2). It was further observed that morphine-exposed macrophages reduce the synthesis and release of monocyte chemotactic protein 1 (MCP-1) and VEGF [58] - both are important mobilizers and attractants for circulating endothelial progenitor cells [86, 87]. Hence, it is attractive to speculate that systemically applied morphine impairs wound vasculogenesis by inhibiting macrophage function. Whether morphine impairs macrophage functions by direct interaction with the immune cells or indirectly by enhancing cortisol concentration *via* activation of the hypothalamic pituitary adrenal axis [88] remains to be evaluated.

## OPIOID EFFECTS ON TUMOR ANGIOGENESIS

Angiogenesis is a prominent hallmark of cancer as it promotes tumor progression at two critical steps: first, it allows and supports tumor growth by providing nutrients and oxygen *via de-novo* formed capillary network [89–91]; and second, it enables metastasis by allocating a route for cancer cells leaving the primary tumor site towards the vascular system [5]. Tumor angiogenesis requires a complex communication between tumor and endothelial cells, cancer-associated fibroblasts and immune cells, which is tightly orchestrated by diverse angiogenic factors including VEGF [92]. The finding that plasma from morphine- and fentanyl-treated breast cancer patients is enriched with VEGF [93] leads to the assumption that opioids could influence tumor angiogenesis. However, *in-vivo* studies that were designed to further clarify the role of opioids in tumor vascularization yielded contradictory observations as both pro-and anti-angiogenic effects were reported.

### Opioids - promoters of tumor angiogenesis

**Figure 3:**
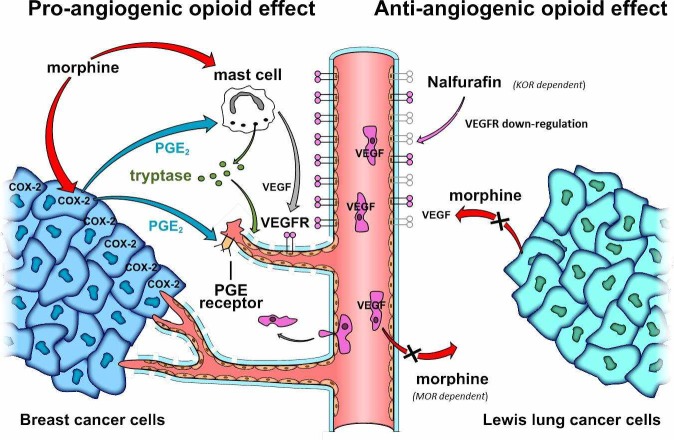
Opiodergic mechanisms of tumor angiogenesis Pro-angiogenic opioid effect (*left panel*): Clinically relevant morphine concentrations induce degranulation of tumor-associated mast cells and release of pro-angiogenic tryptase. Moreover, morphine enhances COX-2 expression and PGE_2_ synthesis in breast cancer cells. PGE2 may further promote vessel formation by inducing VEGF expression in tumor-associated mast cells (“mast cell potentiation”) and by stimulating pro-angiogenic PGE receptor signaling in endothelial cells. Anti-angiogenic opioid effects (*right panel*): High dose morphine impairs VEGF expression in Lewis lung cancer cells, and prevents tumor infiltration by VEGF-producing immune cells in a MOR dependent manner (neutrophils and macrophages/monocytes [*pink*]). Nalfurafine impairs angiogenic signaling by inducing down-regulation of endothelial VEGF receptors *via* KOR stimulation.

Pro-angiogenic opioid effects became evident in a breast cancer xenograft model (using human MCF-7 and MDA-MB-231 cells) where long-term subcutaneous application of therapeutically dosed morphine-sulfate enhanced tumor vascularization [37, 94]. Comparable effects were also observed in allograft tumor models using mammary carcinoma (SCK) cells or Ehrlich mammary adenocarcinoma cells [95, 96]. There, systemic morphine-sulfate treatment increased the density of dilated and branching vessels within the tumor. In line, morphine-sulfate enhanced vascularization in spontaneously grown mammary tumors in transgenic C3TAG mice [41, 97]. Nguyen et al. [97] found that morphine-mediated tumor vascularization in the spontaneous breast cancer model correlated with enhanced degranulation of cancer-associated mast cells and elevated levels of mast cell-specific tryptase within the tumor tissue (Figure 3). As tryptase is a pro-angiogenic protease, which promotes endothelial cell proliferation and tube formation [98], it seems obvious that morphine-triggered release of tryptase from mast cells promotes tumor angiogenesis.

The question remains whether the morphine-triggered tryptase effect is sufficient to initiate tumor vascularization. It seems likely that morphine supports tumor angiogenesis by a further process, such as “mast cell potentiation”. It has been shown that the angiogenic potential of mast cells is boosted by Prostaglandine E_2_ (PGE_2_), which induces VEGF synthesis and release [99]. Farooqui et al. [95] demonstrated that morphine treatment leads to an up-regulation of cyclooxygenase-2 (COX-2) and release of PGE_2_ from mammary tumors (Figure 3). Thus, morphine-triggered tumor vascularization may result from mast cells, which are stimulated for VEGF synthesis and secretion by PGE_2_ released from COX-2 expressing tumor cells. Indeed, the inhibition of COX-2 by celecoxib strongly reduces tumor angiogenesis supporting the hypothesis that COX-2-mediated PGE_2_ synthesis is central to morphine´s angiogenic effect. As an additive effect, PGE_2_ may assist in the induction of proliferation, migration, and tube formation *via* stimulation of PGE_2_ receptor EP3 and FGF receptor transactivation in endothelial cells [100].

### Opioids - inhibitors of tumor angiogenesis

Other *in-vivo* studies report anti-angiogenic opioid effects on tumors. In a Lewis lung carcinoma (LLC) mouse model, tumor vascularization was prevented upon chronic subcutaneous application of morphine-sulfate *via* a MOR-dependent signaling mechanism [53, 57]. Two mechanisms may account for this observation. (i) Morphine is known to inhibit VEGF synthesis and release by interfering with the p38 MAP kinase/HIF-1α signaling axis in LLC cells [57]. Consequently, tumor vascularization may be impaired because of insufficient cancer cell-derived VEGF. (ii) The anti-angiogenic effect of morphine may be also mediated by MOR-expressing host/non-tumor cells. This notion is supported by the fact that the amounts of tumor infiltrating neutrophils and monocyte/macrophage are drastically reduced in morphine-treated mice [53]. As these immune cells are pivotal VEGF donors [101, 102], morphine might impair tumor angiogenesis by interfering with leukocyte transmigration. Indeed, previous *in-vitro* studies demonstrated that the transmigration activity of leukocytes is regulated by MORs and may be blocked by chronic morphine exposure [53]. Transmigration activity involves chemotaxis and cell migration, both of which may be modulated by opioids. Chemotaxis of leukocytes was found to be suppressed by “trans-desensitization” of chemokine receptors in consequence of prolonged MOR and DOR stimulation [103]. The phenomenon goes back to the formation of chemokine receptor heterodimers with MORs and DORs, which are internalized after opioid receptor activation by “sequestrating opioids” such as DAMGO [104]. In contrast to other opioids, morphine is well known to fail MOR and DOR internalization, so that also chemokine receptors would rather remain at the plasma membrane than undergo desensitization and sequestration after morphine exposure [105–107]. Moreover, chronic morphine was shown to prevent internalization of heterologous GPCRs by affecting beta-arrestin function [106]. Thus, dysfunctional chemotaxis rather plays a minor role in impaired leukocyte infiltration after chronic morphine exposure. As an alternative, inhibition of leukocyte migration activity may account for impaired tumor infiltration. Stimulation of leukocyte MORs and DORs has been reported to modulate cell migration in a concentration-dependent manner; whereas high doses prevent, low morphine concentrations promote cell migration [108]. As LLC bearing mice were treated with supratherapeutic morphine concentrations, lack of tumor infiltration is likely to result from high dose inhibition of leukocytes migration activity.

An alternative anti-angiogenic *in-vivo* mechanism was postulated for Nalfurafine, a KOR agonist (Figure 3). Nalfurafine inhibits vascularization of B16 melanomas in mice by inducing VEGF receptor down-regulation in endothelial cells [51]. As Nalfurafine had no effect on tumor angiogenesis in KOR knockout mice, the process requires KOR activity. The mechanism of VEGF receptor down-regulation has not been analyzed in detail yet. One possible explanation could be that VEGF receptors are co-internalized and degraded together with Nalfurafine-stimulated KORs [51, 109]. Alternatively, Nalfurafine may induce VEGF receptor down-regulation by activating PKC-ζ, as it initiates VEGF receptor internalization and degradation by C-tail phosphorylation [110] and represents a KOR-specific down-stream effector [111]. This KOR specific signaling mechanism might also account for the anti-angiogenic morphine effect seen in the LLC tumor model as high morphine concentrations may also bind and stimulate KORs [112].

## OPIOID EFFECTS IN WOUND HEALING AND TUMOR ANGIOGENESIS - THERAPEUTIC RELEVANCE

As angiogenesis plays a central role in the pathogenesis of chronic wounds and cancer, the angiogenic opioid effects may be used as novel therapeutic avenues for these disorders. Impaired angiogenesis, together with the inability of fibroblasts and keratinocytes to proliferate and migrate, are hallmarks of chronic wounds [113]. Topical application of opioids could combat chronic wounds from two different angles. First, opioids may restore wound vascularization. Second, opioids could act beneficially on fibroblast and keratinocyte abnormalities, because they may induce proliferation and migration as well as cytokine production required for wound healing [72, 73, 114]. Indeed, direct application of morphine on painful skin lesions exerted healing effects in two separately treated individuals, but failed to show significant effects in a small group of patients [115–117]. To further test opioids as potential “wound-healing agents”, clinical trials with larger patient groups and special focus on the healing benefit have been designed for topically applied morphine-sulfate and hydromorphine (https://clinicaltrials.gov/; NCT00306020, NCT00177060). Another ongoing trial is testing the topical application of morphine hydrochloride for healing of painful oral lesions (https://eudract.ema.europa.eu; EudraCT Number: 2007-007011-85 8). Moreover, two Phase III studies are currently running with the aspect of wound pain relief by topically applied morphine including wound assessment as a secondary outcome measure (NCT00755989; NCT02028923). In parallel to these clinical studies, there are investigations of new pharmaceutical formulations which should guarantee clinically relevant concentrations of topically applied opioids in wounds. One recently designed innovative drug vehicle are morphine-coated solid nanoparticles, which have been tested in an artificial 3D epidermis skin model [118]. Whereas the *in-vitro* studies revealed a promising wound reepithelization, the nanoparticles still need to be tested in patients.

Systemic application of opioids can support or reduce tumor angiogenesis, depending on the opioid (morphine *vs*. Nalfurafine), the opioid receptor type (MOR *vs*. KOR), the concentration (clinically relevant *vs*. supratherapeutic) and possibly the tumor types (breast *vs*. lung cancer). Angiogenesis leads to tumor growth and metastasis, so that the use of pro-angiogenic drugs is highly critical for tumor patients. Morphine is the opioid of first choice for moderate and severe cancer pain [9]. Thus, the pro-angiogenic morphine effect (seen with clinically relevant concentrations) may be associated with tumor-promoting side effects. This conclusion is supported by epidemiologic studies, which suggested the rate of cancer relapse and metastasis correlates with systemic morphine application in patients with rectal, lung, ovarial adenocarcinoma, prostate or breast cancer [119–123]. As treatment of cancer patients is inconceivable without morphine, two different strategies are currently discussed to circumvent the tumor-promoting side-effect: i) local (epidural, intrathecal, paravertebral) application of morphine, which reduced the incidence of cancer recurrence compared to systemic opioid application [124], ii) co-application with a solely peripheral acting opioid antagonists such as methylnaltrexone, which is already used to prevent peripheral opioid side effects such as obstipation without affecting analgesia [125]. Before these treatment strategies are fully established, the tumor/angiogenesis-promoting opioid effects should be considered a critical side effect.

The central role of angiogenesis for tumor growth and metastasis also leads to the development of anti-angiogenic therapeutics to overcome tumor progression. Some promising therapeutics are the monoclonal antibodies Bevacicumab and Aflibercept, which bind and neutralize VEGF [126], and the small molecular VEGF receptor inhibitors Sunitinib, Sorafenib, Axitinib, and Pazopanib [127]. By inducing down-regulation of VEGF receptors, the KOR agonist Nalfurafine may enhance therapeutic efficiency of the VEGF/VEGF receptor-targeting drugs. In addition, Nalfurafine may be useful to overcome the therapeutic problem of Bevacizumab resistance, which results from enhanced autocrine VEGF/VEGF receptor signaling in response to prolonged Bevacizumab exposure [128]. Despite these promising therapeutic approaches, the safety of Nalfurafine in cancer patients needs to be further tested.

## CONCLUSION

A variety of data, provided by *in-vitro*, animal and clinical studies indicate that opioids modulate angiogenesis. Depending on the opioid receptor type, concentration and application route, opioids act as pro- or anti-angiogenic factors during wound healing and tumor growth. The underlying processes include direct stimulation of endothelial cells, but also of fibroblasts, keratinocytes, immune cells, and tumor cells - triggering the release of angiogenic factors such as NO, PGE_2_, and VEGF. Detailed understanding of further participating factors that form up the opioid-controlled network are needed and indispensable to evaluate the clinical significances of opioids angiogenic effects. It also needs to be mentioned that most of the studies were carried out with morphine. Several opioid effects - including endothelial VEGF receptor activation and vessel formation - were found to be opioid receptor-independent, which points to causal morphine targets beyond the classical opioid receptor signaling cascades. Whether the summarized angiogenic potential and signaling mechanisms are common opioid effects, which could apply to other clinical relevant opioids, has yet to be evaluated.

## Abbreviations

COX: Cyclooxygesase
DAMGO: [D-Ala^2^, N-Me-Phe^4^, Gly^5^-ol]-Enkephalin
DOR: δ-Opioid Receptor
ECSCR: Endothelial Cell-Specific Chemotaxis Receptor
ERK: Extracellular signal regulated kinase
FGF: Fibroblast growth factor
GPCR: G protein-coupled receptor
HDMEC: Human Dermal Microvascular Endothelial Cells
JNK: c-Jun-N-terminal kinase
KOR: k-Opioid Receptor
LLC: Lewis-Lung cell carcinoma
L-NAME: Nω-Nitro-L-arginine methyl ester
LPS: Lipopolysaccharide
miRNA: microRNA
MOR: μ-Opioid Receptor
NO: nitric oxide
Nor-BNI: Norbinaltorphimine
OGFR: Opioid Growth Factor Receptor
PDGF: Platelet Derived Growth Factor
PGE: Prostaglandine E
RNS: Reactive Nitrogen Species
siRNA: small interfering RNA
VEGF: Vascular Endothelial Growth Factor

## References

[1] Flamme I, Frölich T, Risau W (1997). Molecular mechanisms of vasculogenesis and embryonic angiogenesis. J Cell Physiol.

[2] DiPietro L (2012). a. Angiogenesis and scar formation in healing wounds. Curr Opin Rheumatol.

[3] Robich MP, Chu LM, Oyamada S, Sodha NR, Sellke FW (2011). Myocardial Therapeutic Angiogenesis: A Review of the State of Development and Future Obstacles. Expert Rev Cardiovasc Ther.

[4] Neely KA, Gardner TW (1998). Ocular neovascularization: clarifying complex interactions. Am J Pathol.

[5] Folkman J (2002). Role of angiogenesis in tumor growth and metastasis. Semin Oncol.

[6] Crawford T, Alfaro D, Kerrison J, Jablon E (2009). Diabetic retinopathy and angiogenesis. Curr Diabetes Rev.

[7] Stein C, Küchler S (2013). Targeting inflammation and wound healing by opioids. Trends Pharmacol Sci.

[8] Tegeder I, Geisslinger G (2004). Opioids as modulators of cell death and survival—unraveling mechanisms and revealing new indications. Pharmacol Rev.

[9] Hanks G, Conno F, Cherny N, Hanna M, Kalso E, McQuay H, Mercadante S, Meynadier J, Poulain P, Ripamonti C, Radbruch L, Casas J, Sawe J (2001). Morphine and alternative opioids in cancer pain: the EAPC recommendations. Br J Cancer.

[10] Martin C, De Baerdemaeker A, Poelaert J, Madder A, Hoogenboom R, Ballet S (2016). Controlled-release of opioids for improved pain management. Mater Today.

[11] Fioravanti B, Vanderah TW (2008). The ORL-1 receptor system: are there opportunities for antagonists in pain therapy?. Curr Top Med Chem.

[12] Zagon IS, Verderame MF, McLaughlin PJ (2002). The biology of the opioid growth factor receptor (OGFr). Brain Res Rev.

[13] Al-Hasani R, Bruchas MR (2011). Molecular Mechanisms of Opioid Receptor-dependent Signaling and Behavior. Anesthesiology.

[14] Eisinger DA, Ammer H (2011). Epidermal Growth Factor Treatment Switches δ-Opioid Receptor-Stimulated Extracellular Signal-Regulated Kinases 1 and 2 Signaling from an Epidermal Growth. Mol Pharmacol.

[15] Heiss A, Ammer H, Eisinger DA (2009). delta-Opioid receptor-stimulated Akt signaling in neuroblastoma x glioma (NG108-15) hybrid cells involves receptor tyrosine kinase-mediated PI3K activation. Exp Cell Res.

[16] Eisinger DA, Ammer H (2009). Down-regulation of c-Cbl by morphine accounts for persistent ERK1/2 signaling in delta-opioid receptor-expressing HEK293 cells. J Biol Chem.

[17] Olianas MC, Dedoni S, Onali P (2011). Regulation of PI3K/Akt signaling by N-desmethylclozapine through activation of δ-opioid receptor. Eur J Pharmacol.

[18] Schulz R, Eisinger DA, Wehmeyer A (2004). Opioid control of MAP kinase cascade. Eur J Pharmacol.

[19] Gavioli EC, Marzola G, Guerrini R, Bertorelli R, Zucchini S, De Lima TCM, Rae GA, Salvadori S, Regoli D, Calo G (2003). Blockade of nociceptin/orphanin FQ-NOP receptor signalling produces antidepressant-like effects: Pharmacological and genetic evidences from the mouse forced swimming test. Eur J Neurosci.

[20] Filliol D, Ghozland S, Chluba J, Martin M, Matthes HW, Simonin F, Befort K, Gavériaux-Ruff C, Dierich a, LeMeur M, Valverde O, Maldonado R, Kieffer BL (2000). Mice deficient for delta- and mu-opioid receptors exhibit opposing alterations of emotional responses. Nat Genet.

[21] Sora I, Takahashi N, Funada M, Ujike H, Revay RS, Donovan DM, Miner LL, Uhl GR (1997). Opiate receptor knockout mice define mu receptor roles in endogenous nociceptive responses and morphine-induced analgesia. Proc Natl Acad Sci U S A.

[22] Lutz P-E, Kieffer BL (2013). Opioid receptors : distinct roles in mood disorders. Trends Neurosci.

[23] Yan Wu, Mclaughlin PJ, Zagon IS (1998). Ontogeny of the opioid growth factor, [Met5]-enkephalin, preproenkephalin gene expression, and the zeta opioid receptor in the developing and adult aorta of rat. Dev Dyn.

[24] Zagon IS, Wu Y, McLaughlin PJ (1996). Opioid growth factor-dependent DNA synthesis in the neonatal rat aorta. Am J Physiol.

[25] Yamamizu K, Hamada Y, Narita M (2015). k-Opioid receptor ligands regulate angiogenesis in development and in tumours. Br J Pharmacol.

[26] Blebea J, Mazo JE, Kihara TK, Vu JH, McLaughlin PJ, Atnip RG, Zagon IS (2000). Opioid growth factor modulates angiogenesis. J Vasc Surg.

[27] Dai X, Cui SG, Wang T, Liu Q, Song HJ, Wang R (2008). Endogenous opioid peptides, endomorphin-1 and -2 and deltorphin I, stimulate angiogenesis in the CAM assay. Eur J Pharmacol.

[28] Mannelli P, Peindl KS, Wu LT (2011). Pharmacological enhancement of naltrexone treatment for opioid dependence: a review. Subst Abuse Rehabil.

[29] Kobilka BK, Deupi X (2007). Conformational complexity of G-protein-coupled receptors. Trends Pharmacol Sci.

[30] Oldham WM, Hamm HE (2008). Heterotrimeric G protein activation by G-protein-coupled receptors. Nat Rev Mol Cell Biol.

[31] Perez DM, Karnik SS (2005). Multiple signaling states of G-protein-coupled receptors. Pharmacol Rev.

[32] Holmes DIR, Zachary I (2005). The vascular endothelial growth factor (VEGF) family: angiogenic factors in health and disease. Genome Biol.

[33] Robinson CJ, Stringer SE (2001). The splice variants of vascular endothelial growth factor (VEGF) and their receptors. J Cell Sci.

[34] Shibuya M (2011). Vascular Endothelial Growth Factor (VEGF) and Its Receptor (VEGFR) Signaling in Angiogenesis: A Crucial Target for Anti- and Pro-Angiogenic Therapies. Genes Cancer.

[35] Kliche S, Waltenberger J (2001). Critical Review VEGF Receptor Signaling and Endothelial Function. Int Union Biochem Mol Biol Life.

[36] Chen C, Farooqui M, Gupta K (2006). Morphine stimulates vascular endothelial growth factor-like signaling in mouse retinal endothelial cells. Curr Neurovasc Res.

[37] Gupta K, Kshirsagar S, Chang L, Schwartz R, Law P, Yee D, Hebbel R (2002). Morphine Stimulates Angiogenesis by Activating Proangiogenic and Survival-promoting Signaling and Promotes Breast Tumor Growth. Cancer Res.

[38] Singleton PA, Lingen M, Fekete M, Garcia J, Moss J (2006). Methylnaltrexone inhibits opiate and VEGF-induced angiogenesis: Role of receptor transactivation. Microvasc Res.

[39] Jin ZG, Ueba H, Tanimoto T, Lungu AO, Frame MD, Berk BC (2003). Ligand Independent Activation of VEGF Receptor 2 by Fluid Shear Stress Regulates Activation of Endothelial Nitric Oxide Synthase. Circ Res.

[40] Balasubramanian S, Ramakrishnan S, Charboneau R, Wang J, Barke RA, Roy S (2001). Morphine sulfate inhibits hypoxia-induced vascular endothelial growth factor expression in endothelial cells and cardiac myocytes. J Mol Cell Cardiol.

[41] Luk K, Boatman S, Johnson KN, Dudek OA, Ristau N, Vang D, Nguyen J, Gupta K (2012). Influence of morphine on pericyte-endothelial interaction: Implications for antiangiogenic therapy. J Oncol.

[42] Bromann PA, Korkaya H, Courtneidge SA (2004). The interplay between Src family kinases and receptor tyrosine kinases. Oncogene.

[43] Amanchy R, Zhong J, Hong R, Kim JH, Gucek M, Cole RN, Molina H PA (2009). Identification of c-Src tyrosine kinase substrates in platelet-derived growth factor receptor signaling. Mol Oncol.

[44] Klinker JF, Seifert R (1997). Morphine and muscle relaxants are receptor-independent G-protein activators and cromolyn is an inhibitor of stimulated G-protein activity. Inflamm Res.

[45] Pan JB, Ji N, Pan W, Hong R, Wang H, Ji ZJ (2014). High-throughput identification of off-targets for the mechanistic study of severe adverse drug reactions induced by analgesics. Toxicol Appl Pharmacol.

[46] Andrae J, Gallini R, Betsholtz C (2008). Role of platelet-derived growth factors in physiology and medicine. Genes Dev.

[47] Lam CF, Chang PJ, Huang YS, Sung YH, Huang CC, Lin MW, Liu YC, Tsai YC (2008). Prolonged use of high-dose morphine impairs angiogenesis and mobilization of endothelial progenitor cells in mice. Anesth Analg.

[48] Hsiao P, Chang M, Cheng W, Chen C, Lin H, Hsieh C, Sun W (2009). Morphine induces apoptosis of human endothelial cells through nitric oxide and reactive oxygen species pathways. Toxicology.

[49] Pasi A, Qu B, Steiner R, Senn H, Bär W, Messiha F (1991). Angiogenesis: modulation with opioids. Gen Pharmacol.

[50] Stefano GB, Hartman A, Bilfinger TV, Magazine HI, Liu Y, Casares F, Goligorsky MS (1995). Presence of the μ3 Opiate Receptor in Endothelial Cells - coupling of nitric oxide production and vasodilation. J Biol Chem.

[51] Yamamizu K, Furuta S, Hamada Y, Yamashita A, Kuzumaki N, Narita M, Doi K, Katayama S, Nagase H, Yamashita JK, Narita M (2013). Opioids inhibit tumor angiogenesis by suppressing VEGF signaling. Sci Rep.

[52] Szabo I, Chen XH, Xin L, Adler MW, Howard OMZ, Oppenheim JJ, Rogers TJ (2002). Heterologous desensitization of opioid receptors by chemokines inhibits chemotaxis and enhances the perception of pain. Proc Natl Acad Sci U S A.

[53] Koodie L, Yuan H, Pumper JA, Yu H, Charboneau R, Ramkrishnan S, Roy S (2014). Morphine inhibits migration of tumor-infiltrating leukocytes and suppresses angiogenesis associated with tumor growth in mice. Am J Pathol.

[54] Gendelman HE, Ding S, Gong N, Liu L, Ramirez SH, Persidsky Y, Mosley RL, Wang T, Volsky DJ, Xiong H (2009). Monocyte chemitactic protein-1 regulates voltage-gated K+ channels and macrophages transmigration. J Neuroimmune Pharmacol.

[55] Kim JS, Lemasters JJ (2006). Opioid receptor-independent protection of ischemic rat hepatocytes by morphine. Biochem Biophys Res Commun.

[56] Umaru B, Pyriochou A, Kotsikoris V, Papapetropoulos A, Topouzis S (2015). ATP-Sensitive Potassium Channel Activation Induces Angiogenesis in vitro and in vivo. J Pharmacol Exp Ther.

[57] Koodie L, Ramakrishnan S, Roy S (2010). Morphine suppresses tumor angiogenesis through a HIF-1alpha/p38MAPK pathway. Am J Pathol.

[58] Martin JL, Charboneau R, Barke RA, Roy S (2010). Chronic morphine treatment inhibits LPS-induced angiogenesis: Implications in wound healing. Cell Immunol.

[59] Verma A, Bhattacharya R, Remadevi I, Li K, Pramanik K, Samant G V, Horswill M, Chun CZ, Zhao B, Wang E, Miao RQ, Mukhopadhyay D, Ramchandran R (2010). Endothelial cell - specific chemotaxis receptor ( ecscr ) promotes angioblast migration during vasculogenesis and enhances VEGF receptor sensitivity. Blood.

[60] Armstrong LJ, Heath VL, Sanderson S, Kaur S, Beesley JFJ, Herbert JMJ, Legg JA, Poulsom R, Bicknell R (2008). ECSM2, an endothelial specific filamin a binding protein that mediates chemotaxis. Arterioscler Thromb Vasc Biol.

[61] Fang K, Bruce M, Pattillo CB, Zhang S, Stone R, Clifford J, Kevil CG (2011). Temporal genomewide expression profiling of DSS colitis reveals novel inflammatory and angiogenesis genes similar to ulcerative colitis. Physiol Genomics.

[62] Coltrini D, Di Salle E, Ronca R, Belleri M, Testini C, Presta M (2013). Matrigel plug assay: Evaluation of the angiogenic response by reverse transcription-quantitative PCR. Angiogenesis.

[63] Philippe D, Dubuquoy L, Groux H, Brun V, Chuoï-Mariot MT Van, Gaveriaux-Ruff C, Colombel JF, Kieffer BL, Desreumaux P (2003). Anti-inflammatory properties of the mu opioid receptor support its use in the treatment of colon inflammation. J Clin Invest.

[64] Pollet I, Opina CJ, Zimmerman C, Leong KG, Wong F, Karsan A (2003). Bacterial lipopolysaccharide directly induces angiogenesis through TRAF6-mediated activation of NF-kappaB and c-Jun N-terminal kinase. Blood.

[65] Temme S, Jacoby C, Ding Z, Bönner F, Borg N, Schrader J, Flögel U (2014). Technical advance: monitoring the trafficking of neutrophil granulocytes and monocytes during the course of tissue inflammation by noninvasive 19F MRI. J Leukoc Biol.

[66] Banerjee S, Meng J, Das S, Krishnan A, Haworth J, Charboneau R, Zeng Y, Ramakrishnan S, Roy S (2013). Morphine induced exacerbation of sepsis is mediated by tempering endotoxin tolerance through modulation of miR-146a. Sci Rep.

[67] Jin L, Zhao J, Jing W, Yan S, Wang X, Xiao C, Ma B (2014). Role of miR-146a in human chondrocyte apoptosis in response to mechanical pressure injury in vitro. Int J Mol Med.

[68] Wang X, Loram LC, Ramos K, de Jesus AJ, Thomas J, Cheng K, Reddy A, Somogyi AA, Hutchinson MR, Watkins LR, Yin H (2012). Morphine activates neuroinflammation in a manner parallel to endotoxin. Proc Natl Acad Sci.

[69] Mori S, Takada Y (2013). Crosstalk between Fibroblast Growth Factor (FGF) Receptor and Integrin through Direct Integrin Binding to FGF and Resulting Integrin-FGF-FGFR Ternary Complex Formation. Med Sci.

[70] Liu WT, Han Y, Liu YP, Song AA, Barnes B, Song XJ (2010). Spinal matrix metalloproteinase-9 contributes to physical dependence on morphine in mice. J Neurosci.

[71] Gurtner GC, Werner S, Barrandon Y, Longaker MT (2008). Wound repair and regeneration. Nature.

[72] Bigliardi-Qi M, Sumanovski LT, Büchner S, Rufli T, Bigliardi PL (2004). Mu-opiate receptor and beta-endorphin expression in nerve endings and keratinocytes in human skin. Dermatology.

[73] Bigliardi-Qi M, Gaveriaux-Ruff C, Zhou H, Hell C, Bady P, Rufli T, Kieffer B, Bigliardi P (2006). Deletion of delta-opioid receptor in mice alters skin differentiation and delays wound healing. Differentiation.

[74] Gupta M, Poonawala T, Farooqui M, Ericson M, Gupta K (2015). Topical fentanyl stimulates healing of ischemic wounds in diabetic rats. J Diabetes.

[75] Poonawala T, Levay-Young B, Hebbel R, Gupta K (2005). Opioids heal ischemic wounds in the rat. Wound Repair Regen.

[76] Bigliardi PL, Sumanovski LT, Büchner S, Rufli T, Bigliardi-Qi M (2003). Different expression of mu-opiate receptor in chronic and acute wounds and the effect of beta-endorphin on transforming growth factor beta type II receptor and cytokeratin 16 expression. J Invest Dermatol.

[77] Ansel JC, Tiesman JP, Olerud JE, Krueger JG, Krane JF, Tara DC, Shipley GD, Gilbertson D, Usui ML, Hart CE (1993). Human keratinocytes are a major source of cutaneous platelet-derived growth factor. J Clin Invest.

[78] Wang Y, Barker K, Shi S, Diaz M, Mo B, Gutstein HB (2012). Blockade of PDGFR-β activation eliminates morphine analgesic tolerance. Nat Med.

[79] Kurahashi T, Fujii J (2015). Roles of Antioxidative Enzymes in Wound Healing. J Dev Biol.

[80] Howdieshell TR, Webb WL, Sathyanarayana MD, McNeil PL (2003). Inhibition of inducible nitric oxide synthase results in reductions in wound vascular endothelial growth factor expression, granulation tissue formation, and local perfusion. Surgery.

[81] Gerber H, Mcmurtrey A, Kowalski J, Yan M, Keyt B a, Dixit V, Ferrara N (1998). Vascular endothelial growth factor regulates endothelial cell survival through the phosphatidylinositol 3′-kinase/Akt signal transduction pathway. Requirement for Flk-1/KDR activation. J Biol Chem.

[82] McLaughlin PJ, Immonen JA, Zagon IS (2013). Topical naltrexone accelerates full-thickness wound closure in type 1 diabetic rats by stimulating angiogenesis. Exp Biol Med.

[83] Wang D, Sun X, Sadee W (2007). Different effects of opioid antagonists on mu, delta and kappa-opioid receptors with and without agonist pretreatment. J Pharmacol Exp Ther.

[84] Martin JL, Koodie L, Krishnan AG, Charboneau R, Barke RA, Roy S (2010). Chronic morphine administration delays wound healing by inhibiting immune cell recruitment to the wound site. Am J Pathol.

[85] Velazquez OC (2009). Angiogenesis & Vasculogenesis: Inducing the growth of new blood vessels and wound healing by stimulation of Bone Marrow Derived Progenitor Cell Mobilization and Homing. J Vasc Surg.

[86] Urbich C, Dimmeler S (2004). Endothelial progenitor cells: Characterization and role in vascular biology. Circ Res.

[87] Fujiyama S, Amano K, Uehira K, Yoshida M, Nishiwaki Y, Nozawa Y, Jin D, Takai S, Miyazaki M, Egashira K, Imada T, Iwasaka T, Matsubara H (2003). Bone Marrow Monocyte Lineage Cells Adhere on Injured Endothelium in a Monocyte Chemoattractant Protein-1-Dependent Manner and Accelerate Reendothelialization as Endothelial Progenitor Cells. Circ Res.

[88] Baybutt HN, Holsboer F (1990). Inhibition of macrophage differentiation and function by cortisol. Endocrinology.

[89] Folkman J (1971). Tumor angiogenesis: therapeutic implications. N Engl J Med.

[90] Figg WD, Folkman J (2008). Angiogenesis: An integrative approach from science to medicine. Angiogenes An Integr Approach From Sci to Med.

[91] Carmeliet P, Baes M (2008). Metabolism and therapeutic angiogenesis. N Engl J Med.

[92] Weis SM, Cheresh DA (2011). Tumor angiogenesis: molecular pathways and therapeutic targets. Nat Med.

[93] Looney M, Doran P, Buggy DJ (2010). Effect of anesthetic technique on serum vascular endothelial growth factor C and transforming growth factor beta in women undergoing anesthesia and surgery for breast cancer. Anesthesiology.

[94] Bimonte S, Barbieri A, Rea D, Palma G, Luciano A, Cuomo A, Arra C, Izzo F (2015). Morphine Promotes Tumor Angiogenesis and Increases Breast Cancer Progression. Biomed Res Int.

[95] Farooqui M, Li Y, Rogers T, Poonawala T, Griffin RJ, Song CW, Gupta K (2007). COX-2 inhibitor celecoxib prevents chronic morphine-induced promotion of angiogenesis, tumour growth, metastasis and mortality, without compromising analgesia. Br J Cancer.

[96] Ustun F, Durmus-Altun G, Altaner S, Tuncbilek N, Uzal C, Berkarda S (2011). Evaluation of morphine effect on tumour angiogenesis in mouse breast tumour model. EATC. Med Oncol.

[97] Nguyen J, Luk K, Vang D, Soto W, Vincent L, Robiner S, Saavedra R, Li Y, Gupta P, Gupta K (2014). Morphine stimulates cancer progression and mast cell activation and impairs survival in transgenic mice with breast cancer. Br J Anaesth.

[98] Ribatti D, Crivellato E (2012). Mast cells, angiogenesis, and tumour growth. Biochim Biophys Acta - Mol Basis Dis;.

[99] Abdel-Majid RM, Marshall JS (2004). Prostaglandin E2 induces degranulation-independent production of vascular endothelial growth factor by human mast cells. J Immunol.

[100] Finetti F, Solito R, Morbidelli L, Giachetti A, Ziche M, Donnini S (2008). Prostaglandin E2 regulates angiogenesis via activation of fibroblast growth factor receptor-1. J Biol Chem.

[101] Lin EY, Pollard JW (2004). Role of infiltrated leucocytes in tumour growth and spread. Br J Cancer.

[102] Chanmee T, Ontong P, Konno K, Itano N (2014). Tumor-associated macrophages as major players in the tumor microenvironment. Cancers (Basel).

[103] Grimm M, Ben-Baruch A, Taub D, Howard O, Resau J, Wang J, Ali H, Richardson R, Snyderman R, Oppenheim J (1998). Opiates Transdeactivate Chemokine Receptors : δ and μ Opiate Receptor-mediated Heterologous Desensitization. J Exp Med.

[104] Chen C, Li J, Bot G, Szabo I, Rogers TJ, Liu-Chen LY (2004). Heterodimerization and cross-desensitization between the μ-opioid receptor and the chemokine CCR5 receptor. Eur J Pharmacol.

[105] Sternini C, Spann M, Anton B, Keith DE, Bunnett NW, von Zastrow M, Evans C, Brecha NC (1996). Agonist-selective endocytosis of mu opioid receptor by neurons in vivo. PNAS.

[106] Eisinger DA, Ammer H, Schulz R (2002). Chronic morphine treatment inhibits opioid receptor desensitization and internalization. J Neurosci.

[107] Keith DE, Anton B, Murray SR, Zaki P a, Chu PC, Lissin D V, Monteillet-Agius G, Stewart PL, Evans CJ, von Zastrow M (1998). mu-Opioid receptor internalization: opiate drugs have differential effects on a conserved endocytic mechanism in vitro and in the mammalian brain. Mol Pharmacol.

[108] Chadzinska M, Plytycz B (2004). Differential migratory properties of mouse, fish, and frog leukocytes treated with agonists of opioid receptors. Dev Comp Immunol.

[109] Wang Y, Tang K, Inan S, Siebert D, Holzgrabe U, Lee DYW, Huang P, Li JG, Cowan A, Liu-Chen LY (2005). Comparison of pharmacological activities of three distinct kappa ligands (Salvinorin A, TRK-820 and 3FLB) on kappa opioid receptors in vitro and their antipruritic and antinociceptive activities in vivo. J Pharmacol Exp Ther.

[110] Singh AJ, Meyer RD, Band H RN (2005). The carboxyl terminus of VEGFR-2 is required for PKC-mediated down-regulation. Mol Biol Cell.

[111] Belcheva MM, Clark AL, Haas PD, Serna JS, Hahn JW, Kiss A, Coscia CJ (2005). mu and kappa opioid receptors activate ERK/MAPK via different protein kinase C isoforms and secondary messengers in astrocytes. J Biol Chem.

[112] Mignat C, Wille U, Ziegler A (1995). Affinity profiles of morphine, codeine, dihydrocodeine and their glucuronides at opioid receptor subtypes. Life Sci.

[113] Demidova-Rice TN, Durham JT, Herman IM (2012). Wound Healing Angiogenesis: Innovations and Challenges in Acute and Chronic Wound Healing. Adv wound care.

[114] Bigliardi-Qi M, Bigliardi P (2015). The role of opioid receptors in migration and wound recovery in vitro in cultured human keratinocytes and fibroblasts. Methods Mol Biol.

[115] Zaslansky R, Ben-Nun O, Ben-Shitrit S, Ullmann Y, Kopf A, Stein C (2014). A randomized, controlled, clinical pilot study assessing the analgesic effect of morphine applied topically onto split-thickness skin wounds. J Pharm Pharmacol.

[116] Twillman RK, Long TD, Cathers TA, Mueller DW (1999). Treatment of painful skin ulcers with topical opioids. J Pain Symptom Manage.

[117] Watterson G, Howard R, Goldman A (2004). Peripheral opioids in inflammatory pain. Arch Dis Child.

[118] Küchler S, Wolf NB, Heilmann S, Weindl G, Helfmann J, Yahya MM, Stein C, Schäfer-Korting M (2010). 3D-Wound healing model: Influence of morphine and solid lipid nanoparticles. J Biotechnol.

[119] Biki B, Mascha E, Moriarty DC, Fitzpatrick JM, Sessler DI, Buggy DJ (2008). Anesthetic technique for radical prostatectomy surgery affects cancer recurrence: a retrospective analysis. Anesthesiology.

[120] Exadaktylos AK, Buggy DJ, Moriarty DC, Mascha E, Sessler DI (2006). Can anesthetic technique for primary breast cancer surgery affect recurrence or metastasis?. Anesthesiology.

[121] Gupta A, Björnsson A, Fredriksson M, Hallböök O, Eintrei C (2011). Reduction in mortality after epidural anaesthesia and analgesia in patients undergoing rectal but not colonic cancer surgery: A retrospective analysis of data from 655 patients in Central Sweden. Br J Anaesth.

[122] Lin L, Liu C, Tan H, Ouyang H, Zhang Y, Zeng W (2011). Anaesthetic technique may affect prognosis for ovarian serous adenocarcinoma: A retrospective analysis. Br J Anaesth.

[123] Wang K, Qu X, Wang Y, Shen H, Liu Q, Du J (2015). Effect of mu Agonists on Long-Term Survival and Recurrence in Nonsmall Cell Lung Cancer Patients. Medicine (Baltimore).

[124] Lennon FE, Moss J, Singleton P (2012). a. The mu-Opiod Receptor in Cancer progression - Is There a Direct Effect ?. Anesthesiology.

[125] Shaiova L, Rim F, Friedman D, Jahdi M (2007). A review of methylnaltrexone, a peripheral opioid receptor antagonist, and its role in opioid-induced constipation. Palliate Support Care.

[126] Papadopoulos N, Martin J, Ruan Q, Rafique A, Rosconi MP, Shi E, Pyles EA, Yancopoulos GD, Stahl N, Wiegand SJ (2012). Binding and neutralization of vascular endothelial growth factor (VEGF) and related ligands by VEGF Trap, ranibizumab and bevacizumab. Angiogenesis.

[127] Bukowski RM (2012). Third Generation Tyrosine Kinase Inhibitors and Their Development in Advanced Renal Cell Carcinoma. Front Oncol.

[128] Mésange P, Poindessous V, Sabbah M, Escargueil AE, de Gramont A, Larsen AK (2014). Intrinsic bevacizumab resistance is associated with prolonged activation of autocrine VEGF signaling and hypoxia tolerance in colorectal cancer cells and can be overcome by nintedanib, a small molecule angiokinase inhibitor. Oncotarget.

